# Ketone Bodies Are Potential Prognostic Biomarkers in Relapsed/Refractory Diffuse Large B-Cell Lymphoma: Results from the R2-GDP-GOTEL Trial

**DOI:** 10.3390/cancers17030532

**Published:** 2025-02-05

**Authors:** Sara Fernández-Castillejo, Joan Badia, Luís de la Cruz-Merino, Alejandro Martín Garcia-Sáncho, Fernando Carnicero-González, Natalia Palazón-Carrión, Eduardo Ríos-Herranz, Fátima de la Cruz-Vicente, Antonio Rueda-Domínguez, Natividad Martínez-Banaclocha, José Gómez-Codina, Jorge Labrador, Francisca Martínez-Madueño, Núria Amigó, Antonio Salar-Silvestre, Delvys Rodríguez-Abreu, Laura Gálvez-Carvajal, Margarita Sánchez-Beato, Mariano Provencio-Pulla, Maria Guirado-Risueño, Esteban Nogales, Víctor Sánchez-Margalet, Carlos Jiménez-Cortegana, Guillermo Rodríguez-García, Raquel Cumeras, Josep Gumà

**Affiliations:** 1 Translational, Epidemiological and Clinical Oncological Research Group (GIOTEC), Department of Oncology, Institut d’Investigació Sanitària Pere Virgili (IISPV), 43204 Reus, Tarragona, Spain; sara.fernandez@iispv.cat (S.F.-C.); joan.badia@iispv.cat (J.B.); francisca.martinez@salutsantjoan.cat (F.M.-M.); josep.guma@salutsantjoan.cat (J.G.); 2 Institut d’Oncologia de la Catalunya Sud (IOCS), Hospital Universitari Sant Joan de Reus, 43204 Reus, Tarragona, Spain; 3 Cancer Immunotherapy Group, Oncohematology and Genetics Department, Biomedicine Institute of Seville (IBIS)/CSIC, 41013 Seville, Spain; luis.cruz.sspa@juntadeandalucia.es (L.d.l.C.-M.); natalia.palazon.sspa@juntadeandalucia.es (N.P.-C.); esteban.nogales.sspa@juntadeandalucia.es (E.N.); 4 Department of Clinical Oncology, University Hospital Virgen Macarena and School of Medicine, University of Sevilla, 41013 Sevilla, Spain; 5 Department of Hematology, Hospital Universitario de Salamanca, Instituto de Investigación Biomédica de Salamanca (IBSAL), Universidad de Salamanca, 37007 Salamanca, Spain; amartingar@usal.es; 6 CIBER de Cáncer (CIBERONC), Institute of Health Carlos III, 28029 Madrid, Spain; 7 Department of Hematology, San Pedro de Alcántara Hospital, 10003 Cáceres, Spain; fcarnicero@yahoo.es; 8 Department of Hematology, Hospital Universitario Virgen de Valme, 41014 Sevilla, Spain; eriosh@aehh.org; 9 Department of Hematology, Hospital Universitario Virgen del Rocío, 41013 Sevilla, Spain; fatima.cruz.sspa@juntadeandalucia.es (F.d.l.C.-V.); guillermo.rodriguez.sspa@juntadeandalucia.es (G.R.-G.); 10 Department of Clinical Oncology. Hospital Universitario Virgen de la Victoria, 29010 Málaga, Spain; ruedadominguez@uma.es (A.R.-D.); laura.galvez.carvajal.sspa@juntadeandalucia.es (L.G.-C.); 11 Department of Oncology, Dr. Balmis General University Hospital, Alicante Institute for Health and Biomedical Research (ISABIAL), 03010 Alicante, Spain; martinez_natban@gva.es; 12 Department of Clinical Oncology, Hospital Universitario y Politécnico La Fe, 46026 Valencia, Spain; gomez_joscod@gva.es; 13 Department of Hematology, Hospital Universitario de Burgos, 09006 Burgos, Spain; jlabradorg@saludcastillayleon.es; 14 Faculty of Medicine and Health Sciences, Universitat Rovira i Virgili (URV), 43201 Reus, Tarragona, Spain; namigo@biosferteslab.com; 15 Biosfer Teslab, 43206 Reus, Tarragona, Spain; 16 Department of Hematology, Hospital del Mar, 08003 Barcelona, Spain; asalar@hospitaldelmar.cat; 17 Department of Clinical Oncology, Hospital Universitario Insular de Gran Canaria, 35016 Las Palmas de Gran Canaria, Las Palmas, Spain; drodabr@gobiernodecanarias.org; 18 Lymphoma Research Group, Department of Medical Oncology, Hospital Universitario Puerta de Hierro-Majadahonda, IDIPHISA, 28222 Majadahonda, Madrid, Spain; msbeato@idiphim.org; 19 Department of Clinical Oncology, Hospital Universitario Puerta De Hierro-Majadahonda, IDIPHISA, 28222 Majadahonda, Madrid, Spain; mariano.provencio@uam.es; 20 Department of Clinical Oncology, Hospital Universitario de Elche, 03203 Elche, Alicante, Spain; mguiradorisu@gmail.com; 21 Medical Biochemistry and Molecular Biology and Immunology, Hospital Universitario Virgen de la Macarena, 41009 Sevilla, Spain; margalet@us.es (V.S.-M.); cjcortegana@us.es (C.J.-C.); 22 Department of Electrical and Automatic Electronic Engineering, Universitat Rovira i Virgili (URV), 43002 Tarragona, Spain

**Keywords:** ketone bodies, diffuse large B-cell lymphoma (DLBCL), relapsed/refractory lymphoma, 3-hydroxybutyrate, 3OHB, acetone, prognostic biomarkers, metabolomics

## Abstract

**Background:** Patients with relapsed or refractory (R/R) diffuse large B-cell lymphoma (DLBCL) who are ineligible for high-dose chemotherapy have limited treatment options and poor life expectancy. The purpose of this study is to identify a serum metabolomic profile that may be predictive of outcome in patients with R/R-DLBCL. **Methods:** This study included 69 R/R DLBCL patients from the R2-GDP-GOTEL trial (EudraCT 2014-001620-299). Serum samples were collected at baseline, and the mean length of follow-up was 41 months. Serum metabolites were analyzed by nuclear magnetic resonance (NMR). Metabolites were correlated with treatment response, progression-free survival (PFS), and overall survival (OS). **Results:** Serum levels of 3-hydroxybutyrate (3OHB) and acetone were significantly (*p* < 0.001) associated with PFS (3OHB: hazard ratio [HR] 7.7, 95% confidence interval [CI] 2.5–24.1; acetone: HR 9.32, 95% CI 2.75–31.6) and OS (3OHB: HR 9.32, 95% CI 2.75–31.6; acetone: HR 1.92, 95% CI 1.36–2.69). Serum values of 141 µM for 3OHB and 40 µM for acetone were the optimal cutoffs associated with the survival outcomes. Elevated 3OHB levels (>141 μM) were specific to the ABC subtype of DLBCL, while acetone levels were elevated in both types of DLCBL but more pronounced in ABC cases. In a multivariate survival analysis, including the International Prognostic Index (IPI) score and refractoriness status (R/R), 3OHB and acetone remained significant. To aid oncologists employing the R2-GDP regime, we constructed PFS and OS nomograms for R/R-DLBCL risk stratification, incorporating 3OHB levels or acetone levels, IPI score, and refractoriness status. The nomogram with 3OHB and refractoriness status showed a time-dependent AUC of 0.86 for 6-month PFS and 0.84 for 12-month OS. These nomograms provide a comprehensive tool for individualized risk assessment and treatment optimization. **Conclusions:** The ketone bodies 3OHB and acetone are potential prognostic biomarkers of poor outcome in R/R DLBCL patients treated with the R2-GDP regimen, independently of IPI score and chemorefractoriness status.

## Simple Summary

Patients with relapsed or refractory diffuse large B-cell lymphoma (DLBCL) have poor outcomes and limited treatment options. A phase II trial by GOTEL evaluated the R2-GDP regimen (combination of lenalidomide, rituximab, gemcitabine, dexamethasone, and cisplatin), demonstrating feasibility and effectiveness. Baseline serum metabolomic analysis of 69 patients enrolled in the trial identified two independent metabolites, 3-hydroxybutyrate (3OHB) and acetone, as being significantly associated with overall survival and progression-free survival. Elevated 3OHB levels (>141 μM) were specific to the ABC subtype of DLBCL, while acetone levels were elevated in both types of DLCBL but more pronounced in ABC cases. These biomarkers, irrespective of sex, age, and BMI, could help predict outcomes and guide treatment strategies in relapsed/refractory DLBCL.

## 1. Introduction

Approximately 60% of patients with diffuse large B-cell lymphomas (DLBCL) are cured using upfront therapy with the CHOP-R regimen or other anthracycline and rituximab-based chemotherapies, whereas the remaining 40% are refractory or relapsed (R/R) following first-line chemotherapy. At the time this clinical trial was conducted, the standard treatment for R/R DLBCL patients was second-line conventional chemotherapy followed by consolidation with high-dose chemotherapy in chemosensitive patients. Palliative chemotherapy is an option for patients who are unable to receive high-dose chemotherapy or CAR-T therapy; nevertheless, the best therapeutic option may involve enrolling the patient in investigational clinical trials.

The identification of biomarkers capable of predicting the outcome of DLBCL patients has been the focus of increasing interest due to the marked genetic and molecular heterogeneity that underlies disease aggressiveness and tumor progression. Metabolomics is a powerful tool that can identify cancer biomarkers and drivers of tumorigenesis. In the field of lymphomas, different studies have evaluated untargeted metabolomics using gas (GC) or liquid chromatography (LC) coupled with mass spectrometry (MS) in patients with lymphoid neoplasms and healthy populations [1,2,3,4,5]. Although most studies showed metabolomic differences between patients and healthy subjects, the identification of differential metabolites has been inconsistent. This is likely due to differences in laboratory techniques (GC-MS and LC-MS) and/or biological samples (blood, urine, and feces). However, the metabolomic profile in patients with DLBCL as a prognostic factor for survival has been evaluated in only two studies. Stenson et al. [6] used nuclear magnetic resonance (NMR) spectroscopy in 87 patients with DLBCL prior to starting first-line treatment with chemoimmunotherapy. Statistically significant differences were found in the metabolomic profile between patients who achieved a complete response and long survival and those who were refractory to treatment or relapsed. Patients who had been cured showed higher levels of aspartate, valine, ornithine, and pyroglutamate, whereas R/R patients had higher concentrations of lysine, arginine, cadaverine, and 2-hydroxybutyrate. In a second study, Mi et al. [7] used GC-MS to assess pre-treatment serum samples from 80 DLBCL patients and reported that higher levels of pyroglutamic and hexadecenoic acids and lower levels of valine were associated with significantly higher overall survival.

The Spanish Group for the Treatment and Study of Lymphomas (GOTEL) conducted a phase II clinical trial to evaluate the combination of lenalidomide with R-GDP (rituximab, gemcitabine, dexamethasone, and cisplatin) in patients with R/R DLBCL who were either unsuitable for high-dose chemotherapy or whose treatment had not worked. A total of 78 patients were included in the R2-GDP-GOTEL study, and after a median follow-up of 37 months, 7.9% of patients were still alive without progression at 24 months [8]. Taking advantage of baseline data from this clinical trial, the present study was designed to identify a serum metabolomic profile that may be predictive of outcomes in patients with R/R DLBCL treated with the R2-GDP combination.

## 2. Materials and Methods

### 2.1. Study Design and Patients

The R2-GDP-GOTEL clinical trial was a phase II, multicenter, open-label, and single-arm study carried out in 78 R/R DLBCL patients who were treated in 18 Spanish hospitals between April 2015 and September 2018. Briefly, the R2-GDP regimen included an induction treatment with a combination of lenalidomide, rituximab, gemcitabine, dexamethasone, and cisplatin (R2-GDP), for up to 6 cycles (every 3 weeks), followed by maintenance with lenalidomide for up to 24 months, unless there was a progression, unacceptable toxicity, or voluntary withdrawal [8]. Additional information about the dropout rate (attrition rate) and a power analysis of the R2-GDP trial have been previously reported [8]. Since this trial has a single-arm design, randomization and blinding do not apply. Eligible participants were patients with chemorefractory or relapsed DLBCL unsuitable for high-dose chemotherapy, with an Eastern Cooperative Oncology Group (ECOG) performance status of 0–1, and who had previously received at least one line of immunochemotherapy, including rituximab. Patients with leptomeningeal or central nervous system (CNS) involvement, and those with hematological, renal, or liver dysfunction, were excluded from the initial R2-GDP-GOTEL clinical trial. Since all the lymphomas included in this study were DLBCL, all the included patients expressed CD45 and the pan B-cell markers CD19, CD20, CD22, and CD79a in the initial diagnostic immunohistochemical study.

The objective of the present substudy was to assess the baseline metabolomic profile of R/R DLBCL patients and to identify serum metabolites predictive of outcome. For that purpose, 69 patients from the R2-GDP-GOTEL clinical trial for whom a sufficient blood sample was available for metabolomic profiling were included in the metabolomic profiling substudy.

### 2.2. Sample Preparation and Metabolomic Profiling

A comprehensive methodological approach for high-throughput screening by NMR spectroscopy using a Bruker Avance 600 MHz NMR spectrometer (Bruker BioSpin, Ettlingen, Germany) was used to analyze a broad spectrum of metabolites in serum samples [9]. The analysis included the lipoprotein, glycoprotein, and metabolite profiles from intact serum, in addition to the lipid profile from lipid serum extracts obtained by a biphasic extraction with methyl tert-butyl ether (MTBE). Lipid extracts were dried and reconstituted in 0.01% tetramethylsilane (TMS) solution (0.067 mM) and deuterated solvents. All analyses were carried out at Biosfer Teslab (Reus, Tarragona, Spain). In addition, 1D Nuclear Overhauser Effect Spectroscopy (NOESY) was used to characterize small molecules such as amino acids and small carbohydrates, while larger molecules like lipoproteins and glycoproteins were detected using LED Diffusion (Diff) experiments. All the sequences ran at 37 °C in quantitative conditions. Samples were coded to maintain the subjects’ anonymity.

The Liposcale^®^ test (IVD-CE) was used to determine the lipid composition, particle size, and concentration of major lipoprotein classes as well as the particle concentration of nine subclasses [10]. Circulating glycoproteins were obtained by deconvoluting the specific NMR spectral regions and quantifying areas correlating to the concentration of the acetyl groups of N-acetylglucosamine and N-acetyl galactosamine (GlycA) and acetyl groups of N-acetylneuraminic acid (GlycB). A Carr–Purcell–Meiboom–Gill (CPMG) filter on the 1H-NMR spectra was used to profile and absolutely quantify the metabolomics profile. The BUME protocol [11] was used for lipid quantification, based on the Lipspin software [12]. Succinctly, we used lineshape fitting analysis of spectral regions to quantify the lipids.

### 2.3. Endpoints

The primary endpoints were the characterization of the baseline metabolomic profile in R/R DLBCL patients according to response to treatment and outcome. Key secondary endpoints were to determine the predictive performance of nomograms for R/R DLBCL risk stratification based on the metabolites identified and their optimal cutoffs associated with the outcome. Tumor response was evaluated according to the International Working Group Criteria [13] using computed tomography (CT) after the third induction cycle and positron emission tomography (PET) in the following 4 weeks after the last cycle of the induction phase. Outcome included progression-free survival (PFS) and overall survival (OS). PFS was defined as the time between the first dose of the R2-GDP schedule to the progression of disease or death, and OS was defined as the period from the first dose of the R2-GDP schedule to death from any cause.

### 2.4. Statistical Analysis

Statistical analyses were performed with R software using the R Stats Package (v.4.3.2.), survival (v.3.5-7), survminer (v.0.4.9), maxstat (v.0.7-25), caret (v.6.0-94), rms (v.6.7-1), and survivalROC (v.1.0.3.1). The analyses were restricted to metabolites identified in >90% of patients. Metabolite missing values were not imputed, and metabolite concentrations were only scaled in multivariate analyses. For reproducibility, the random seed was set at 123 via the set.seed function (R base). Univariate statistics was performed using the non-parametric Mann–Whitney–Wilcoxon statistical test. Survival analysis was carried out using the non-parametric Kaplan–Meier method and the log-rank test for the comparison of survival curves. Cutoffs for numerical variables were calculated using the maximally selected rank statistics, with a 10% minimum proportion of observations, and missing values were assigned to the lowest group. Cox regression analyses were run twice: once to search for statistically significant individual prognostic metabolites, and again to confirm whether they remained statistically significant in a multivariate analysis with other statistically significant clinical prognostic factors, such as the International Prognostic Index (IPI) [14] and refractoriness status. The hazard ratio (HR) and the 95% confidence interval (CI) were calculated. Statistical significance was set at *p* < 0.05, and a false discovery rate (FDR) *p* value adjustment was applied when necessary. Fold change (FC) analyses were used to compare the absolute value of change in the means of each metabolite between responders and non-responders.

The nomogram for R/R DLBCL risk stratification incorporated the significant metabolites identified in the univariate Cox analysis and the significant clinical risk factors within a Cox proportional hazards framework. A 70% train split was used for model fitting via bootstrap calibration (B = 1000), while the remaining 30% test split was used for predictive performance evaluation with a time-dependent receiver operating characteristic curve (ROC) and area under the ROC (AUC). The time points selected were 6 months for PFS and 12 months for OS. Pearson’s product-moment correlation coefficient (*r*) was used to assess the relationship between the metabolites identified.

## 3. Results

### 3.1. Baseline Characteristics of Patients and Survival

Sixty-nine participants in the R2-GDP-GOTEL clinical trial (35 men and 34 women) with a median age of 70 years were included in the metabolomic profiling study. Key baseline data are displayed in Table 1. Activated B-cell-like (ABC) lymphomas, relapsed DLBCL, and IPI low/high-intermediate risk category (0–3) were the most common characteristics.

In this subset of patients, the overall response to R2-GPD treatment was 59.4% (*n* = 41) (complete response 37.7%, partial response 21.7%), similar to those previously reported in the whole R2-GDP population [8]. The individual clinical evolution of the 69 patients is presented in Supplementary Figure S1. After a median follow-up of 41 months, the median PFS was 5 months (36%, 16%, and 7.9% at 6, 12, and 24 months, respectively) and the median OS was 12 months (66%, 47%, and 36% at 6, 12, and 24 months, respectively) (Supplementary Figure S2A,B). Survival analyses stratified by cell-of-origin (CoO) showed a median PFS of 6.0 months (95% CI 3.0–11) for the germinal center B-cell-like (GBC) subtype and 5.0 months (95% CI 2–6) for ABC (*p* = 0.099) (Figure S2C), similar to those previously reported in the whole R2-GDP population [8]. The median OS was 16 months (95% CI 6.2—not reached) for GBC and 9.3 months (95% CI 5.3–24) for ABC (*p* = 0.59) (Figure S2D). Concerning DLBCL status, patients with chemorefractory disease showed a median PFS of 3 months (95% CI 2–6), whereas those with relapsed disease had a median PFS of 6 months (95% CI 4–9) (*p* = 0.083) (Figure S2E). The OS was significantly longer for the relapsed group (median 24 months, 95% CI 12—not reached) than for the chemorefractory group (median 6.2 months, 95% CI 4.5–13) (*p* = 0.0034) (Figure S2F). Patients in the low/high-intermediate-risk IPI category showed a median PFS of 6 months (95% CI 4–9), whilst patients in the high-risk category had a median PFS of 2 months (95% CI 2–7) (*p* = 0.0044) (Figure S2G). The median OS was also longer for the low/high-intermediate-risk IPI category (median 15 months, 95% CI 9.3–33) compared with the high-risk IPI category (median 3.5 months, 95% CI 1.8—not reached) (*p* = 0.074) (Figure S2H).

### 3.2. Treatment Response Metabolomic Profile

A total of 66 metabolites were identified, 7 of which were excluded as they were detected in less than 90% of the patients. In Supplementary Table S1, the mean values for the treatment response of the 59 included metabolites are presented. Twelve metabolites were significantly different (*p* < 0.05) in responders vs. non-responders, including creatinine, lactate, glycoprotein A, free cholesterol, esterified cholesterol, intermediate-density lipoprotein (IDL) cholesterol, IDL triglycerides, high-density lipoprotein (HDL) triglycerides, medium and large low-density lipoproteins (LDLs), and medium and large HDLs. All metabolites except for creatinine showed significantly lower mean values in responders to R2-GPD treatment than in non-responders. By contrast, creatinine was significantly higher in responders than in non-responders (Table 2). The box plots for significant metabolite among responders and non-responders are shown in Supplementary Figure S3.

### 3.3. Survival Outcome and Metabolomic Profile

**Figure 1:**
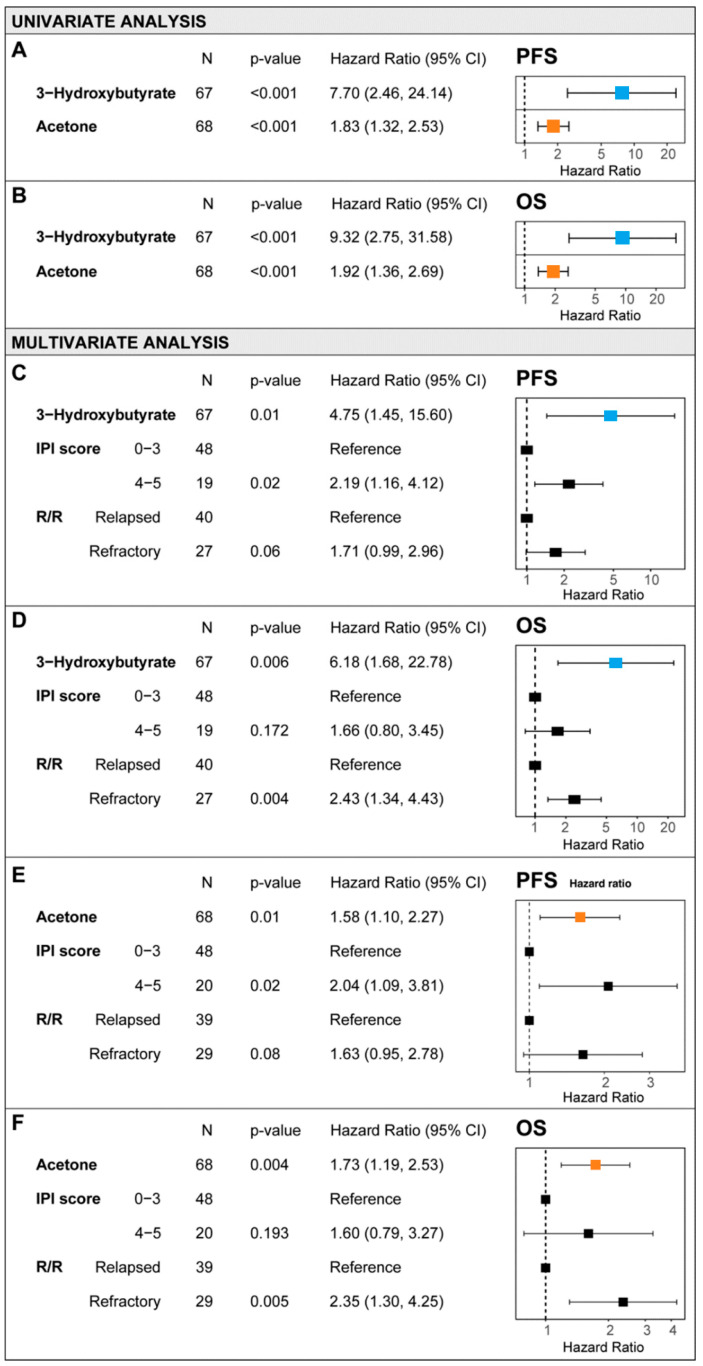
3-Hydroxybutyrate and acetone as prognostic metabolites in the Cox univariate analysis (**A**,**B**) and multivariate regressions models (**C**–**F**) for progression-free survival (PFS) (**A**,**C**,**E**) and overall survival (OS) (**B**,**D**,**F**).

Two metabolites, 3OHB and acetone, were significantly associated with survival outcomes in the Cox univariate regression analysis. Higher serum concentrations of 3OHB or acetone were statistically significant (*p* < 0.001) prognostic factors for a worse PFS (3OHB: hazard ratio [HR] = 7.7; acetone: HR = 1.83) (Figure 1A) and OS (3OHB: HR = 9.32; acetone: HR = 1.92) (Figure 1B). Both 3OHB and acetone were independent predictors that were significantly associated with PFS and OS in the multivariate Cox regression model, which included IPI risk categories and refractoriness status (R/R) as classical DLBCL clinical prognostic factors (Figure 1C–F). In the overall study population, the mean serum levels of 3OHB were 120.6 μM (range 0–1455.9 μM) (*n* = 67) and the mean levels of acetone 40.48 μM (range 0–534.2 μM) (*n* = 68).

**Figure 2:**
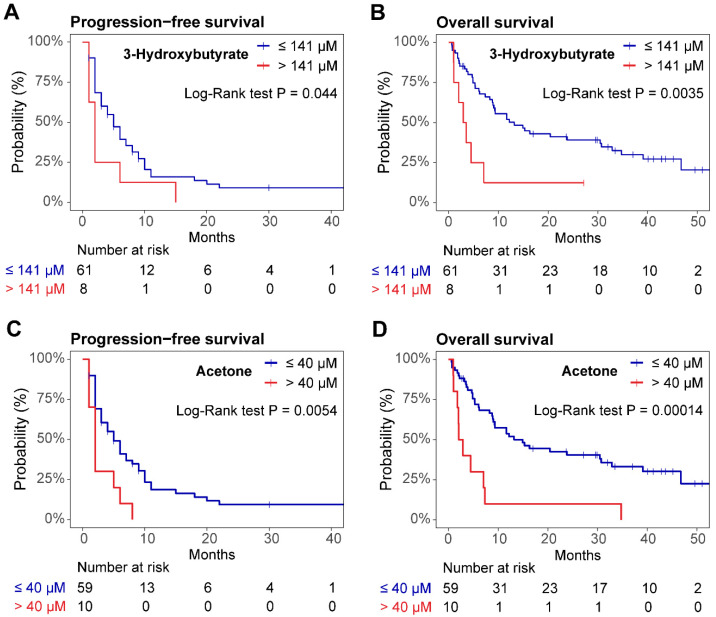
Kaplan–Meier survival curves. (**A**,**B**), progression-free survival (PFS) and overall survival (OS) for the cutoff of 3-hydroxybutyrate (3OHB); (**C**,**D**) PFS and OS for the cutoff of acetone.

The optimal cutoffs for serum 3OHB and acetone were identified with an outcome-oriented method (see Methods) and were set at 141 µM and 40 µM, respectively. Following the application of the cutoff, the PFS and OS Kaplan–Meier survival curves were significantly different. For serum 3OHB, the median PFS was 5 months (95% CI 3–8) vs. 2 months (95% CI 1.0—not reached) (*p* = 0.044) (Figure 2A). The corresponding values for OS were 13 months (95% CI 8.9–33) vs. 3.2 months (95% 2.0—not reached) (*p* = 0.0035), respectively (Figure 2B). Differences in PFS for serum acetone were 5 months (95% CI 3–9) vs. 2 months (95% CI 1.0—not reached) (*p* = 0.0054) (Figure 2C) and 15 months (95% CI 9–33) vs. 2.5 months (95% CI 1.8—not reached) (*p* = 0.00014) for OS, respectively (Figure 2D). Significant differences were also observed in univariate Cox regressions following the application of the cutoff. For serum 3OHB, the HR for PFS was 2.21, while the HR for OS was 3.21. For serum acetone, the HR for PFS was 2.76 and the HR for OS was 3.71 (Supplementary Figure S4).

### 3.4. Nomogram for R/R DLBCL Risk Stratification

**Figure 3:**
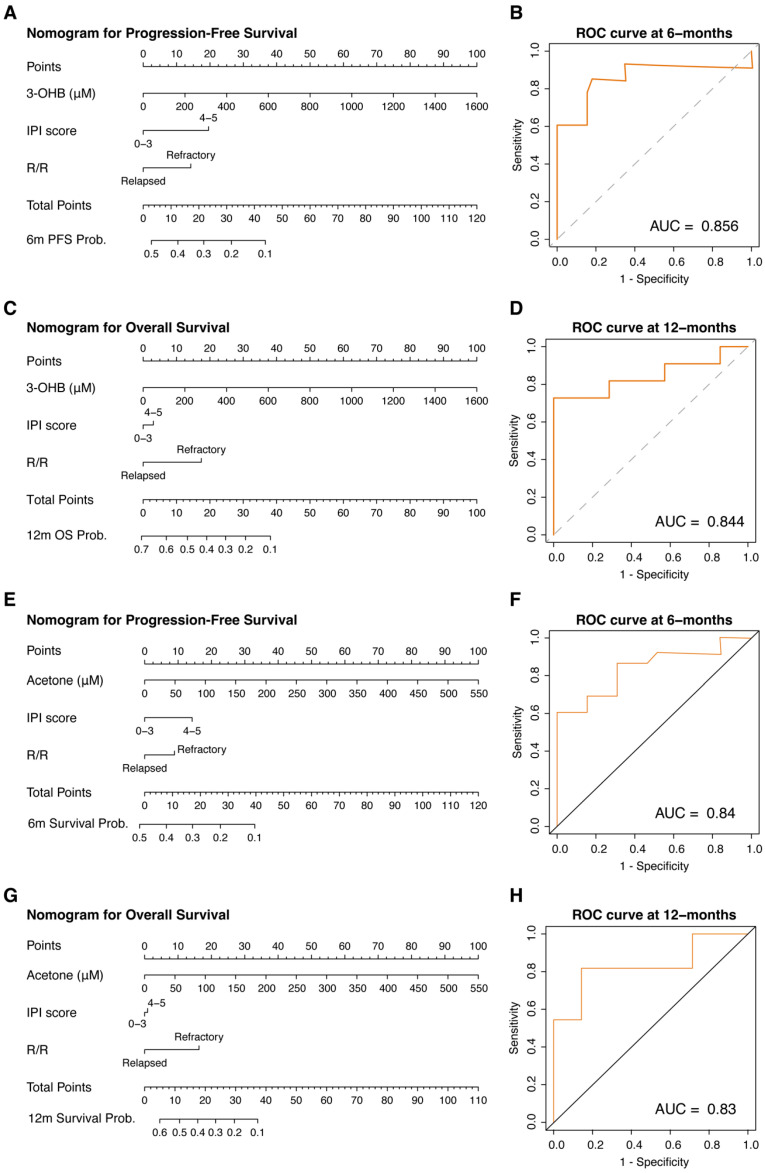
Nomograms for R/R DLBCL risk stratification based on single metabolites and their respective time-dependent ROCs. (**A**,**B**) 3-Hydroxybutyrate (3OHB) nomogram for progression-free survival (PFS) and its ROC; (**C**,**D**) 3OHB nomogram for overall survival (OS) and its ROC; (**E**,**F**) acetone nomogram for PFS and its ROC; (**G**,**H**) acetone nomogram for OS and its ROC.

Nomograms for risk stratification in patients with R/R DLBCL based on serum metabolites 3OHB and acetone showed a high predictive performance, slightly more favorable for 3OHB, with an AUC of 0.856 for 6-month PFS (Figure 3A,B) and 0.844 for 12-month OS (Figure 3C,D). Serum acetone showed a similar predictive performance, with an AUC of 0.840 for 6-month PFS (Figure 3D,E) and 0.830 for 12-month OS (Figure 3F,G). A nomogram that included both 3OHB and acetone was also explored; however, it performed worse (data not shown) since both metabolites showed a statistically significant correlation (*r* = 0.76, *p* < 0.001) (Supplementary Figure S5). Let us consider the OS nomogram of 3OHB for two patients: Patient 1 and Patient 2. Although both patients have an IPI score of 4–5 (3 points) and are both refractory (18 points), Patient 1 has a 3OHB concentration of 800 µM (50 points), whereas Patient 2 has a 3OHB concentration of 50 µM (3 points). Hence, for the 3OHB OS nomogram, Patient 1 scored 71 points (<10% survival probability at 12 months), while Patient 2 scored 24 points (30% survival probability at 12 months).

## 4. Discussion

The present data contribute to defining the metabolomic profile linked to R/R-DLBCL patients’ responsiveness to treatment and survival. To the best of our knowledge, no previous metabolomics study has been reported in this setting. Treatment respondents had lower levels of free and esterified cholesterol, a lesser number of triglycerides in IDL and LDL, a lower content of cholesterol in IDL, and an overall reduction in medium and small LDL and HDL particle numbers than non-responders. These findings suggest lower transportation of triglycerides and cholesterol to extrahepatic tissues. Regarding survival analysis, we identified two new prognostic factors for PFS and OS, the ketone bodies 3OHB and acetone. These factors were independent of other known clinical factors such as IPI score, chemorefractoriness, and CoO. Kaplan–Meier’s survival curves showed that the optimal cutoff (based on *p*-values) was 141 µM for 3OHB and 40 µM for acetone. However, 3OHB and acetone were not significantly different when comparing responders vs. non-responders to R2-GDP treatment. Nonetheless, based on the obtained results for PFS and OS, increased baseline serum concentrations of 3OHB and acetone could be promising biomarkers of an adverse prognosis.

**Figure 4:**
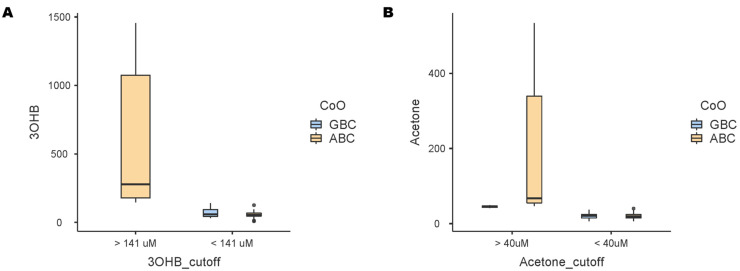
Ketone bodies of a poor prognosis per cell-of-origin (CoO) subtypes: germinal center B-cell-like (GBC) and activated B-cell-like (ABC) lymphomas. (**A**) 3-Hydroxybutyrate (3OHB) boxplot per concentration cutoff values (141 μM) and CoO. (**B**) Acetone boxplot per concentration cutoff values (40 μM) and CoO.

Even though responses did not differ between GCB and ABC DLCBL in the R2-GDP schedule [8], further analysis of baseline 3OHB and acetone concentrations showed that they might be indicative of poor prognosis per molecular subtype for DLCBL. In fact, only activated B-cell-like lymphomas show high levels of 3OHB, which are associated with a bad prognosis (>141 μM) (Figure 4). On the other hand, while elevated levels of acetone are found in both types of DLCBL, the ABC-type lymphomas exhibit the highest concentrations. Therefore, elevated levels of 3OHB and acetone could be specific biomarkers of poor prognosis in ABC DLCBL. Also, 3OHB and acetone are independent of anthropomorphic data (sex, age, and BMI), as shown by a correlation analysis heatmap (Supplementary Figure S6).

Metabolomic reprogramming in cancer cells is widely regarded as a cancer hallmark, including changes in glycolysis, glutaminolysis, choline metabolism, and fatty acid β-oxidation rates [15,16,17,18]. DLBCL cells favoring glycolysis are resistant to hypoxic stress through global translational repression and a decreased mitochondrial function, mediated by hypoxia-inducible factor 1-α (HIF-1α) [19], in line with the Warburg effect [20]. However, under acidotic conditions, tumor cells favor HIF-2α-mediated glutaminolysis and fatty acid β-oxidation over glycolysis [21,22]. DLBCLs with low glyceraldehyde-3-phosphate dehydrogenase (GADPH) primarily rely on oxidative phosphorylation (OxPhos), through mTORC1 signaling and glutaminolysis, and show poorer responses to R-CHOP-based therapies [23]. OxPhos-DLBCLs also show poor response to histone deacetylase inhibitors (HDACis), needing a combination with choline pathway inhibitors [24] or antioxidant production inhibitors [25] to rescue sensitivity. Targeting both glycolysis and OxPhos simultaneously has also been proposed as a potential therapeutic strategy [26].

3OH, acetone, and acetoacetate are ketone bodies synthesized from acetyl-CoA produced by fatty acid β-oxidation, which occurs mainly in the liver but also in the kidneys and placenta. They are then transported to extrahepatic tissues to be used in the mitochondrial oxidative phosphorylation process, which is facilitated by the enzyme 3-oxoacid CoA-transferase 1 (OXCT1), and they then enter the tricarboxylic acid cycle (TCA) to obtain energy [27,28,29]. Acetoacetate and 3OHB are the predominant ketonic bodies, while acetone, the smallest ketone body, is in equilibrium with acetoacetate and undergoes spontaneous transformation. 3OHB is synthesized from acetoacetate via the liver enzyme 3-hydroxybutyrate dehydrogenase (BDH1) [27]. Ketogenesis occurs in the mitochondria of hepatic cells as an alternative fuel source during episodes of energy restriction, such as fasting and physical exercise, which prompts a shift towards the β-oxidation of free fatty acids from the adipose tissue. Moreover, hepatic ketogenesis is inhibited by insulin and activated by glucagon and ketogenic amino acids as a response to ensure whole-body energy supply.

Recently, BDH1 has been identified as a significant predictor of poor survival outcomes in DLBCL patients [30], suggesting a shared mechanism of metabolic dysregulation associated with adverse outcomes. 3OHB, acetone, and BDH1 play integral roles in energy metabolism pathways. 3OHB and acetone, key ketone bodies, and BDH1, an enzyme regulating ketone body metabolism, may reflect systemic metabolic stress or tissue damage, which could underlie their association with survival. Elevated 3OHB and acetone may reflect increased ketogenesis or impaired utilization of ketone bodies, while high BDH1 levels suggest enhanced metabolic demands or stress in tissues, potentially contributing to disease progression. Caro et al. found that OXCT1 and Acetyl-CoA Acetyltransferase (ACAT1, also known as acetoacetyl Coenzyme A thiolase) were increased in the mitochondria proteome of a metabolomic subtype of DLBCL, the OxPhos-DLBCL subtype patients [31]. Taken together, these results suggest we have identified a metabolomic subtype of DLBCL that utilizes ketone bodies as one of the substrates for oxidative phosphorylation. This fact highlights the metabolomic heterogeneity in DLBCL that may be of importance for survival and treatment refractoriness. Nonetheless, the adverse prognosis of R/R-DLBCL patients with elevated 3OHB and acetone may be reflecting systemic metabolic stress, other clinical comorbidities, or altered drug metabolism, such as the cisplatin resistance conferred by an increase in aldo–keto reductases [32]. Pharmacokinetic studies in cisplatin-based therapies may explain the mechanistic basis for the adverse prognosis associated with elevated serum 3OHB and acetone in R/R-DLBCL.

Circulating ketone body levels in healthy adults are around 200 μM, but these concentrations increase to 1 mM after a few hours of fasting and vigorous exercise [28] and up to 5–7 mM after prolonged starvation [33]. However, little is known regarding blood concentrations of ketone bodies in cancer patients. In the present study, 3OHB was detected in 97% of patients and acetone in 98.5%, with median values of 120.6 μM and 40.48 μM, respectively. Ketone acetoacetate quantification was not possible with the technology used in the study. In this study, it is worthy of note that blood samples were obtained after an overnight fast, so the observed differences in ketonic body levels in our population cannot be attributed to differences in fasting conditions.

Both serum levels of 3OHB and acetone were significantly associated with a worse prognosis in R/R DLBCL patients. In line with our results, ketone bodies are known to promote tumor growth [28]. In a study of breast cancer cell lines expressing monocarboxylate transporter 2 (MCT2), treatment with β-hydroxybutyrate enhanced tumorigenic properties by upregulating the transcription of tumor-promoting genes [34]. Additionally, ketone catabolism was found to be reactivated in hepatocellular carcinoma cells under deprivation conditions, suggesting a metabolic adaptation process by which these cells employ ketone bodies for energy supply and cancer progression during nutrient deprivation [29]. In a tissue metabolomic study, greater levels of 3OHB were observed in grade 3 endometrial cancer as compared to grade 1 tumors and healthy tissue [35], and in early-stage breast cancer patients (FIGO stages I and II), increased levels of 3OHB were also reported [36]. In a study of pancreatic ductal adenocarcinoma tumor cells, β-hydroxybutyrate was proposed as an alternative cell-intrinsic system promoting adenocarcinoma tumor cell growth and progression in conditions of oxygen and nutrient deprivation [37]. Taken together, 3OHB-related inhibition of class I histone deacetylases (HDACs) may induce the upregulation of tumor-promoting genes through the epigenetic modification of chromatin [28,34], and metabolic adaptations of cancer cells may facilitate the use of 3OHB as an alternative energy source through the activation of OXCT1 expression [28,29].

It has been reported that 3OHB may exert antineoplastic effects by modulating cellular signaling pathways. In addition, cancer cells seem to be unable to use ketone bodies for energy due to their acquired metabolic inflexibility [28]. In this sense, other studies have reported reduced levels of 3OHB in colorectal [38] and pancreatic cancer patients [39,40]. 3OHB was shown to exhibit anti-colorectal cancer activity through G protein-coupled 109A (GPR109A, also known as hydroxyl-carboxylic acid receptor 2; HCAR2) binding and activation [38], a tumor suppressor that inhibits lipolysis in the adipocytes [28]. Moreover, some central nervous system tumors have low expression of OXCT1 and BDH1 enzymes, suggesting an inability of central nervous system tumors to utilize ketone bodies as energy substrates [28]. Furthermore, ketone bodies may accumulate in the blood as well as inside DLBCL tumor cells in patients with low levels of blood ketone metabolism-promoting enzymes (such as aldo–keto reductase), slowing down the metabolism of cisplatin and other drugs [40]. Therefore, after R2-GDP therapy, cisplatin metabolism may be delayed, weakening the antitumor effect and worsening the prognosis.

There is limited evidence of altered metabolic biomarkers in B-cell lymphomas [41,42]. To the best of our knowledge, only two other metabolomic studies searched for prognostic biomarkers in DLBC patients. Stenson et al. [6] and Mi et al. [7] measured serum metabolomics in untreated DLBCL patients, through NMR and GC-MS techniques. The Swedish study of 87 patients found a signature for DLBCL with a high risk of failing immunochemotherapy. Briefly, patients with long-term PFS had higher levels of aspartate, valine, ornithine, and pyroglutamate, while R/R patients had higher levels of lysine, arginine, cadaverine, and 2-hydroxybutyrate. Additionally, age-adjusted IPI had a relatively lower impact on the response prognosis [6]. In the Chinese study [7] based on data from 80 patients, higher levels of pyroglutamic and hexadecenoic acid were associated with a better survival rate, while higher levels of valine were linked to a worse survival rate. In the present R2-GDP clinical study, NMR serum metabolomics in R/R DLBCL patients identified 3OHB and acetone as biomarkers of poor prognosis, which suggests a metabolic reprogramming of cancer cells to utilize ketonic bodies as an alternative fuel source. While individuals in the current study had undergone at least one previous chemotherapy line, metabolomic tests were conducted on untreated patients in these two previous studies.

Regarding the tumor microenvironment, Huang et al. [34] showed that 3OHB secreted by mammary gland-derived adipocytes present in the tumor microenvironment penetrates the interior of breast cancer cells expressing MCT2. There, 3OHB increases histone H3K9 and H3K14 acetylation upregulating tumor-promoting genes. Our group previously reported increased plasma levels of fatty acid-binding proteins (FABPs) 4 and 5 in breast cancer patients, suggesting an increased fatty acid transport from peritumoral adipocytes to breast cancer cells [43,44]. Moreover, intracellular levels of FABP4 in MCF-7 and MDA-MB-231 breast cancer cell lines increased after exogenous FABP4 incubation, which enhanced their proliferation rate [45]. These results might point to crosstalk between peritumoral adipocytes and breast cancer cells, which receive 3OHB and fatty acids from the peritumoral adipocytes. The transfer of energy-rich metabolites such as 3OHB and L-lactate, which fuels oxidative phosphorylation in breast cancer cells, was another reported example of the interaction between cancer-associated fibroblasts and breast cancer cells [46].

Currently, CAR-T cells and bispecific antibodies have become the most active treatment options in patients with R/R DLBCL who are ineligible for high-dose chemotherapy. At the time of our study, the R-GDP regimen and other cytostatics, whether used individually or in combination, constituted the standard treatment in this setting. Although the metabolomic prognostic factors identified in our study cannot be strictly extrapolated to other therapeutic modalities, they can serve as working hypotheses for future research. In this regard, our group has initiated a study on multi-omic prognostic factors (including metabolomics) in patients newly diagnosed with DLBCL. If these results are confirmed, the metabolomic profile could be integrated into a prognostic index that encompasses clinical, pathological, genomic, and metabolomic variables in previously untreated patients. Refining such a prognostic index holds significant potential for optimizing patient selection in trials involving new therapies.

This study presents some limitations. First, there were no data taken into account regarding the patients’ comorbidity or concomitant treatment, two variables that could act as confusion factors. Second, since OXCT1, BDH1, and GPR109A levels were not assessed due to lack of hepatic tissue in our study, no mechanistic hypothesis could have been formulated.

## 5. Conclusions

In R/R DLBCL patients treated with R2-GDP, the ketone bodies 3OHB and acetone were statistically significant metabolic biomarkers of poor prognosis. The association between increased serum concentrations of both biomarkers and outcome remained significant after adjusting for IPI score, CoO, and chemorefractoriness. Optimal cutoffs for the best discriminating ability were >141 µM/L for 3OHB and >40 µM/L for acetone. A proposed nomogram may be useful in risk stratification in R/R DLBCL patients. Further studies in untreated DLBCL patients based on a multi-omics approach are necessary to enhance the role of these promising biomarkers in the clinical setting.

## Figures and Tables

**Table 1:** Key characteristics of the study population.

Variables ^1^	Total Patients (*n* = 69)
Sex, *n* (%)	
Men	35 (50.7%)
Women	34 (49.3%)
Age, years, median (IQR)	70.1 (61.7–74.5)
Body mass index (BMI), kg/m^2^, median (IQR)	27.0 (23.9–30.2)
Cell-of-origin (CoO), *n* (%) [*n* = 65]	
Germinal center B-cell-like	27 (41%)
Activated B-cell-like	39 (59%)
Refractoriness, *n* (%)	
Relapsed	40 (58.0%)
Chemorefractory	29 (42.0%)
International Prognostic Index (IPI) score, *n* (%)	
Low risk (0–1)	12 (17.4%)
Low/High-intermediate risk (2–3)	37 (53.6%)
High risk (4–5)	20 (29.0%)
Response, *n* (%)	
Responders	41 (59.4%)
Non-responders	28 (40.6%)

^1^ IQR: interquartile range (25th–75th percentile).

**Table 2:** Significant serum metabolites in responders and non-responders.

Metabolites ^1^	Responders (*n* = 41)	Non-Responders (*n* = 28)	*p* Value	Fold Change ^2^
Low molecular weight				
Creatinine, µM, mean (SD)	73.0 (43.3)	48.7 (17.6)	0.030	1.50
Lactate, µM, mean (SD)	1174.2 (989.7)	2023.5 (1655.6)	0.034	0.58
Glycoproteins				
Glycoprotein A, µmol/L, mean (SD)	846.0 (165.3)	915.5 (161.0)	0.028	0.92
Glycoprotein A, H/W ratio, mean (SD)	24.7 (4.3)	27.04 (4.7)	0.024	0.92
Cholesterol				
Free cholesterol, mmol/L, mean (SD)	2.36 (0.71)	2.84 (0.83)	0.016	0.83
Esterified cholesterol, mmol/L, mean (SD)	4.76 (1.20)	5.36 (1.40)	0.043	0.89
Lipoproteins				
IDL cholesterol, mg/dL, mean (SD)	14.14 (5.84)	17.39 (5.61)	0.015	0.86
IDL triglycerides, mg/dL, mean (SD)	13.38 (4.59)	15.64 (4.07)	0.018	0.81
LDL triglycerides, mg/dL, mean (SD)	18.94 (6.40)	22.90 (6.08)	0.005	0.83
Medium LDL-P, nmol/L, mean (SD)	340.83 (134.98)	424.09 (157.02)	0.008	0.80
Large LDL-P, nmol/L, mean (SD)	180.15 (44.78)	201.32 (51.39)	0.043	0.89
Medium HDL-P, µmol/L, mean (SD)	10.18 (1.68)	11.27 (1.72)	0.006	0.90
Large HDL-P, µmol/L, mean (SD)	0.30 (0.05)	0.33 (0.05)	0.005	0.90

^1^ SD: standard deviation; H/W: height/width of the NMR peak; IDL: intermediate-density lipoprotein; LDL: low-density lipoprotein; HDL: high-density lipoprotein; P: particle number. ^2^ Fold change (FC) > 1 indicates an increase in metabolite concentration and those with FC < 1 indicate a decrease in responders vs. non-responders.

## Data Availability

The de-identified data generated in this study are available within the article and its supplementary data files. Raw data can be obtained upon reasonable request from the corresponding authors.

## Supplementary Materials

The following supporting information can be downloaded at https://www.mdpi.com/article/10.3390/cancers17030532/s1: Figure S1. Clinical evolution of each of the 69 diffuse large B-cell lymphoma patients included in the R2-GDP cohort; Figure S2. Kaplan–Meier survival curves for progression-free survival (PFS) and overall survival (OS). A, PFS for the overall study patients; B, OS for the overall study patients; C and D, stratified by the cell of origin (germinal center B-cell-like [GBC] and activated B-cell-like [ABC]); E and F, refractory lymphoma vs. relapsed lymphoma; G and H, IPI scores 0–3 vs. 4–5; Table S1. Mean values and standard deviation (SD) of metabolites and lipids in responders and non-responders; Figure S3. Box plots of the significantly changed metabolites in responders vs. non-responders; Figure S4. Univariate Cox regression for 3-hydroxybutyrate and acetone after applying their optimal cutoffs, for A progression-free survival (PFS) and B overall survival (OS); Figure S5. Pearson correlation between 3-hydroxybutyrate and acetone; Figure S6. Pearson correlation heatmap analysis of 3-hydroxybutyrate (3OHB), acetone, and anthropomorphic data (age, sex, and body mass index [BMI]).
