# Supplementary material for: Ketone Bodies Are Potential Prognostic Biomarkers in Relapsed/Refractory Diffuse Large B-Cell Lymphoma: Results from the R2-GDP-GOTEL Trial
Source: Cancers (Basel). 2025 Feb 5;17(3):532. doi: 10.3390/cancers17030532 (PMC11817199; doi:10.3390/cancers17030532)
Supplement: Supplementary file 1 [file cancers-17-00532-s001.zip › cancers-3379311-supplementary.pdf]

SUPPLEMENTARY DATA AND MATERIALS

Ketone Bodies Are Potential Prognostic Biomarkers in Relapsed/Refractory Diffuse Large B-Cell Lymphoma: Results from the R2-GDP-GOTEL Trial

Sara Fernández-Castillejo [1,2,†], Joan Badia [1,2,†], Luís de la Cruz-Merino [3,4], Alejandro Martín Garcia-Sáncho [5,6], Fernando Carnicero-González [7], Natalia Palazón-Carrión [3,4], Eduardo Ríos-Herranz [8], Fátima de la Cruz-Vicente [9], Antonio Rueda-Domínguez [10], Natividad Martínez-Banaclocha [11], José Gómez-Codina [12], Jorge Labrador [13], Francisca Martínez-Madueño [1,2,14], Núria Amigó [14,15], Antonio Salar-Silvestre [16], Delvys Rodríguez-Abreu [17], Laura Gálvez-Carvajal [10], Margarita Sánchez-Beato [18], Mariano Provencio-Pulla [19], Maria Guirado-Risueño [20], Esteban Nogales [3,4], Víctor Sánchez-Margalet [21], Carlos Jiménez-Cortegana [21], Guillermo Rodríguez-García [9], Raquel Cumeras [1,22,*] and Josep Gumà [1,2,14] on behalf of the Spanish Lymphoma Oncology Group (GOTEL)

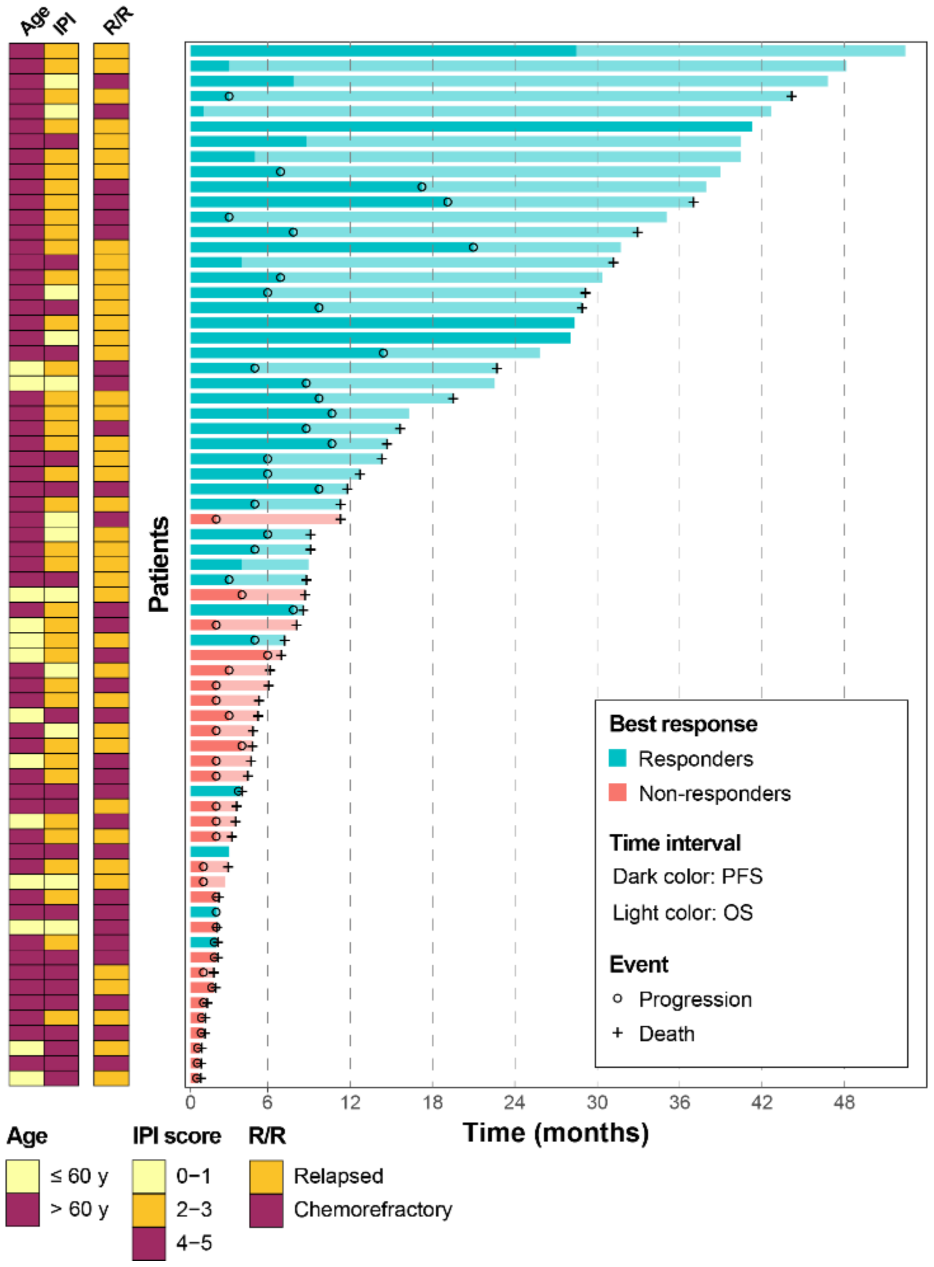

Figure S1. Clinical evolution of each of the 69 diffuse large B-cell lymphoma patients included in the R2-GDP cohort.

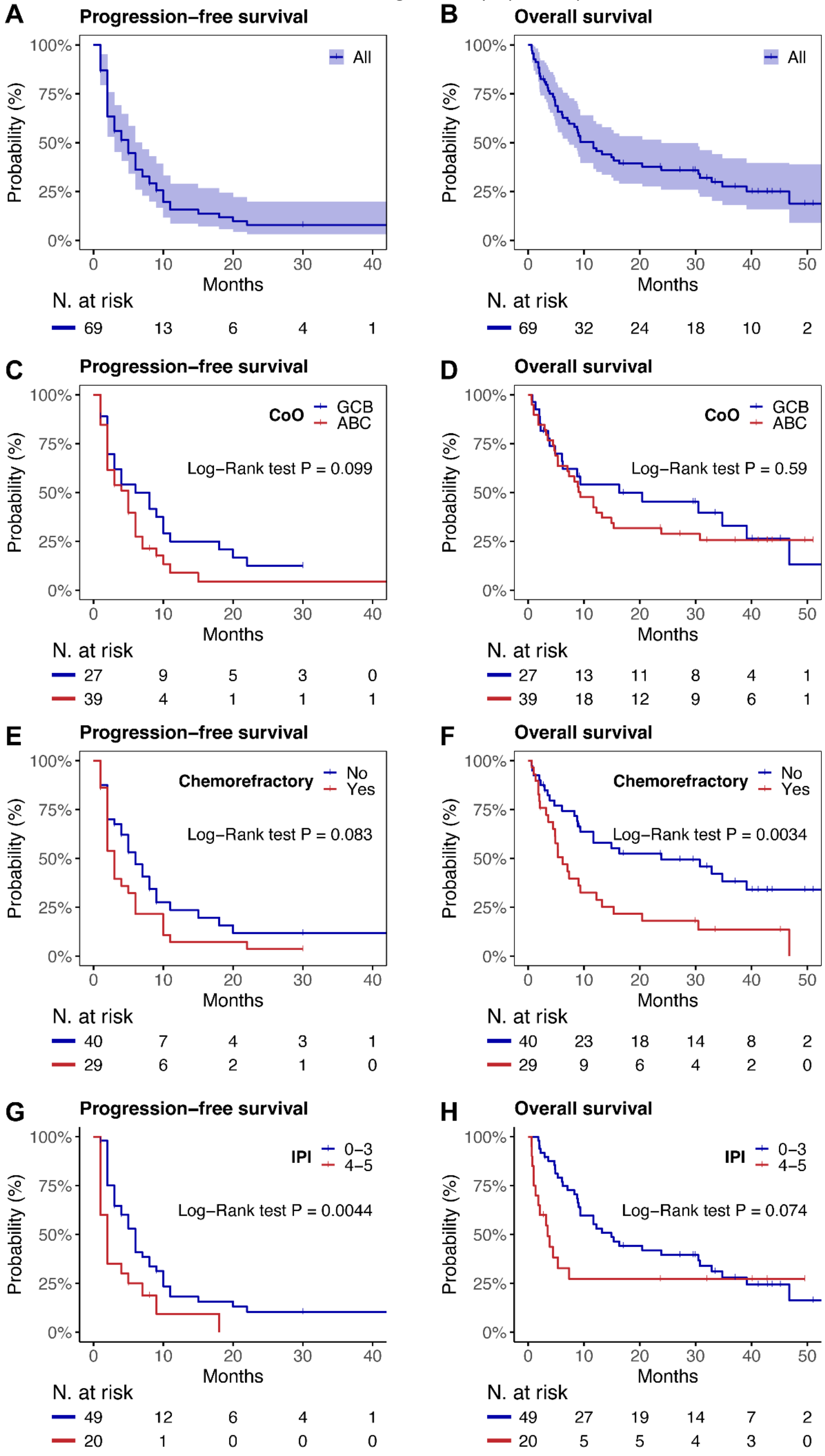

Figure S2. Kaplan-Meier survival curves for progression-free survival (PFS) and overall survival (OS). **A**, PFS for the overall study patients; **B**, OS for the overall study patients; **C** and **D**, stratified by cell of origin (germinal center B-cell-like [GBC] and activated B-cell-like [ABC]); **E** and **F**, refractory lymphoma vs relapsed lymphoma; **G** and **H**, IPI scores 0-3 vs 4-5.

Table S1. Mean values and standard deviation (SD) of metabolites and lipids in responders and non-responders.

**Metabolites**	**Responders**	**Non-responders**	**Total**
Total N (%)	41 (59.4)	28 (40.6)	69
Low molecular weight			
3-Hydroxybutyrate, μM, mean (SD)	69.6 (51.5)	177.4 (324.4)	111.9 (211.2)
Acetate, μM, mean (SD)	24.7 (12.8)	61.6 (119.5)	39.2 (76.7)
Acetone, μM, mean (SD)	59.2 (54.7)	73.5 (76.7)	64.8 (63.9)
Alanine, μM, mean (SD)	361.4 (90.1)	405.9 (108.8)	378.9 (99.3)
Creatinine, μM, mean (SD)	73.0 (43.3)	48.7 (17.6)	63.5 (37.2)
Glucose, μM, mean (SD)	4604.0 (1388.9)	4233.5 (1511.9)	4458.5 (1436.5)
Glutamate, μM, mean (SD)	207.6 (77.2)	237.0 (102.5)	219.1 (88.3)
Glycine, μM, mean (SD)	271.8 (57.8)	275.9 (79.3)	273.4 (66.4)
Histidine, μM, mean (SD)	82.3 (25.7)	81.3 (27.4)	81.9 (26.1)
Lactate, μM, mean (SD)	1174.2 (989.7)	2023.5 (1655.6)	1507.9 (1345.2)
Tyrosine, μM, mean (SD)	48.9 (16.5)	53.4 (13.3)	50.7 (15.4)
Valine, μM, mean (SD)	192.5 (40.4)	196.5 (44.0)	194.1 (41.5)
Isoleucine, μM, mean (SD)	35.5 (13.3)	37.5 (13.2)	36.3 (13.2)
Leucine, μM, mean (SD)	119.5 (52.6)	145.0 (69.9)	129.6 (60.7)
Glycoproteins			
Glyc-B, umol/L, mean (SD)	452.7 (73.2)	478.4 (75.3)	463.1 (74.6)
Glyc-F, umol/L, mean (SD)	266.3 (50.2)	276.3 (42.1)	270.4 (47.0)
Glyc-A, umol/L, mean (SD)	846.0 (165.3)	915.5 (161.0)	874.2 (166.0)
Glyc-B, H/W ratio, mean (SD)	5.7 (0.9)	6.0 (0.9)	5.8 (0.9)
Glyc-A, H/W ratio, mean (SD)	24.7 (4.3)	27.0 (4.7)	25.7 (4.6)
Cholesterol			
FC, mmol/L, mean (SD)	2.4 (0.7)	2.8 (0.8)	2.6 (0.8)
EC, mmol/L, mean (SD)	4.8 (1.2)	5.4 (1.4)	5.0 (1.3)
Lipoproteins			
VLDL-C, mg/dL, mean (SD)	23.7 (11.6)	25.8 (11.4)	24.6 (11.5)
IDL-C, mg/dL, mean (SD)	14.1 (5.8)	17.4 (5.6)	15.5 (5.9)
LDL-C, mg/dL, mean (SD)	118.5 (35.5)	132.9 (38.8)	124.3 (37.3)
HDL-C, mg/dL, mean (SD)	44.5 (14.6)	51.5 (17.5)	47.3 (16.1)
VLDL-TG, mg/dL, mean (SD)	81.6 (41.5)	87.6 (39.9)	84.1 (40.7)
IDL-TG, mg/dL, mean (SD)	13.4 (4.6)	15.6 (4.1)	14.3 (4.5)
LDL-TG, mg/dL, mean (SD)	18.9 (6.4)	22.9 (6.1)	20.5 (6.5)
HDL-TG, mg/dL, mean (SD)	18.3 (8.2)	18.9 (5.6)	18.5 (7.2)
VLDL-P, mg/dL, mean (SD)	60.4 (29.5)	65.0 (28.7)	62.3 (29.0)
largeVLDL-P, nmol/L, mean (SD)	1.6 (0.6)	1.6 (0.6)	1.6 (0.6)
mediumVLDL-P, nmol/L, mean (SD)	6.7 (3.4)	7.2 (3.1)	6.9 (3.3)
smallVLDL-P, nmol/L, mean (SD)	52.2 (26.2)	56.2 (25.7)	53.8 (25.9)
LDL-P, nmol/L, mean (SD)	1204.2 (315.8)	1346.0 (360.7)	1261.7 (339.4)
largeLDL-P, nmol/L, mean (SD)	180.2 (44.8)	201.3 (51.4)	188.7 (48.4)
mediumLDL-P, nmol/L, mean (SD)	340.8 (135.0)	424.1 (157.0)	374.6 (149.0)
smallLDL-P, nmol/L, mean (SD)	683.2 (171.2)	720.6 (186.9)	698.4 (177.3)

Metabolites	Responders	Non-responders	Total
HDL-P, nmol/L, mean (SD)	24.8 (6.9)	26.9 (7.9)	25.6 (7.3)
largeHDL-P, umol/L, mean (SD)	0.3 (0.0)	0.3 (0.0)	0.3 (0.0)
mediumHDL-P, umol/L, mean (SD)	10.2 (1.7)	11.3 (1.7)	10.6 (1.8)
smallHDL-P, umol/L, mean (SD)	14.3 (6.6)	15.3 (7.7)	14.7 (7.1)
VLDL-Z, nm, mean (SD)	42.2 (0.2)	42.2 (0.2)	42.2 (0.2)
LDL-Z, nm, mean (SD)	21.2 (0.2)	21.3 (0.3)	21.2 (0.2)
HDL-Z, nm, mean (SD)	8.4 (0.2)	8.5 (0.3)	8.4 (0.3)
Other lipids			
TG, mmol/L, mean (SD)	2.2 (1.0)	2.3 (1.0)	2.3 (1.0)
PL, mmol/L, mean (SD)	4.5 (1.2)	4.5 (1.4)	4.5 (1.3)
PC, mmol/L, mean (SD)	4.5 (1.3)	4.3 (1.3)	4.4 (1.3)
SM, mmol/L, mean (SD)	1.2 (0.2)	1.2 (0.3)	1.2 (0.3)
LPC, mmol/L, mean (SD)	1.3 (0.4)	1.1 (0.4)	1.2 (0.4)
PUFA1, mmol/L, mean (SD)	2.3 (1.3)	2.3 (1.4)	2.3 (1.3)
PUFA2, mmol/L, mean (SD)	4.9 (2.1)	5.1 (2.4)	5.0 (2.2)
PUFA3, mmol/L, mean (SD)	3.3 (1.7)	3.6 (1.6)	3.4 (1.6)
PUFA4, mmol/L, mean (SD)	3.0 (1.5)	2.6 (1.5)	2.9 (1.5)
Linoleic, mmol/L, mean (SD)	3.8 (1.1)	4.0 (1.0)	3.9 (1.1)
SFA, mmol/L, mean (SD)	8.5 (2.4)	7.8 (2.2)	8.2 (2.4)
w6+w7, mmol/L, mean (SD)	3.9 (1.6)	4.5 (1.4)	4.1 (1.5)
w9, mmol/L, mean (SD)	5.0 (1.2)	5.0 (1.2)	5.0 (1.2)
w3, mmol/L, mean (SD)	0.5 (0.3)	0.5 (0.2)	0.5 (0.2)
ARA+EPA, mmol/L, mean (SD)	0.6 (0.2)	0.6 (0.2)	0.6 (0.2)

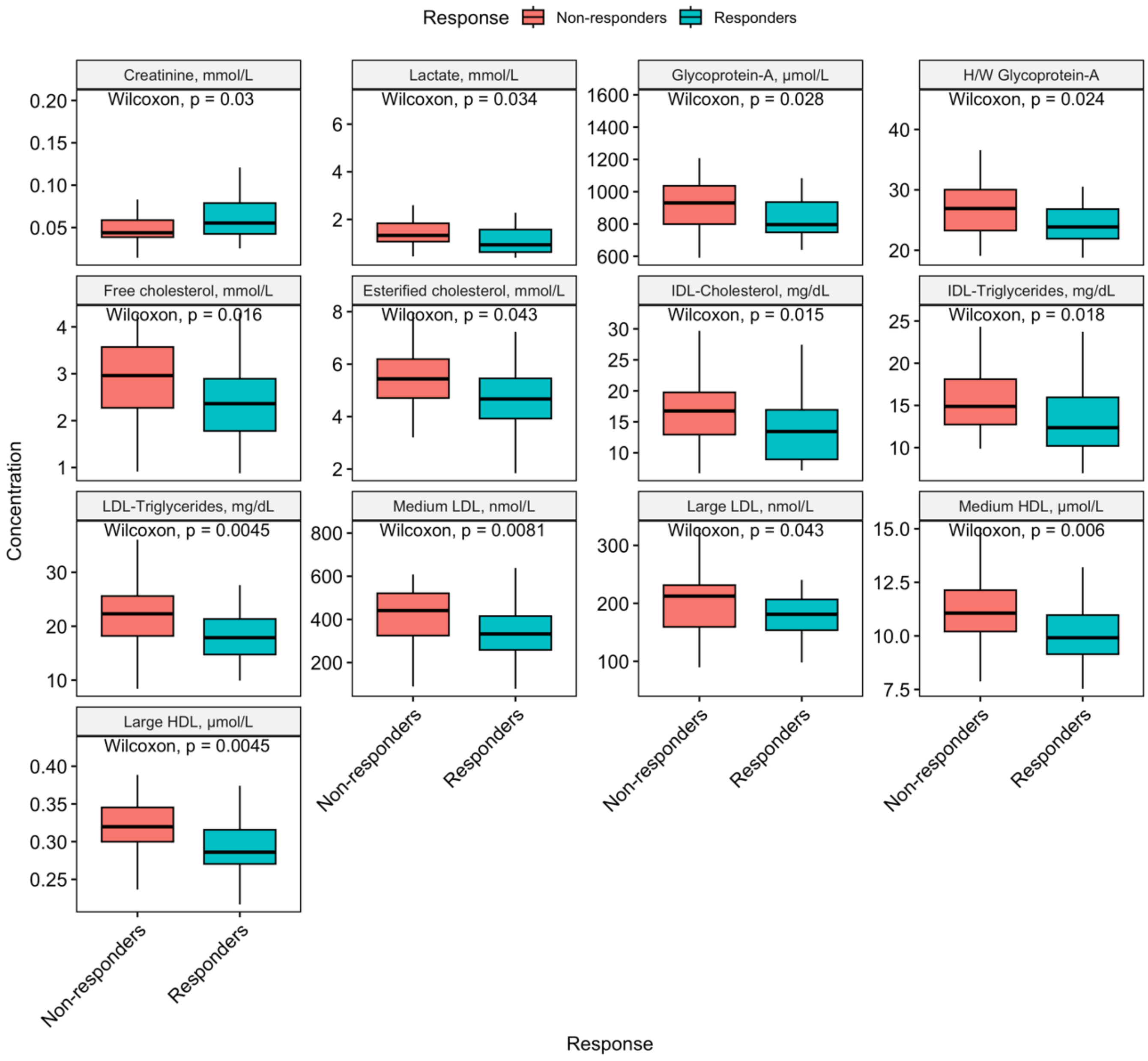

Figure S3. Box plots of the significantly changed metabolites in responders *vs*. non-responders.

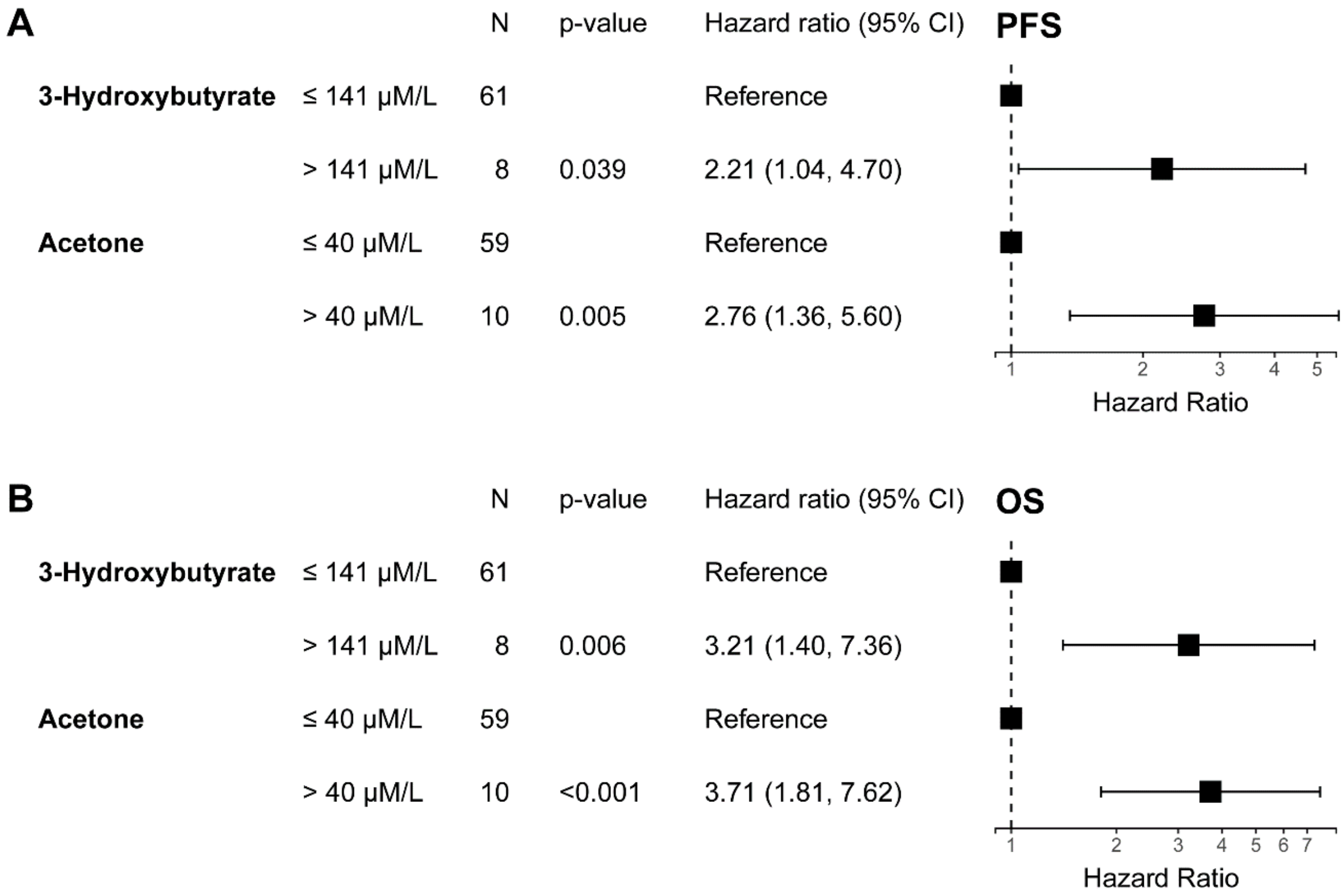

Figure S4. Univariate Cox regression for 3-hydroxybutyrate and acetone after applying their optimal cutoffs, for **A** progression free survival (PFS) and **B** overall survival (OS).

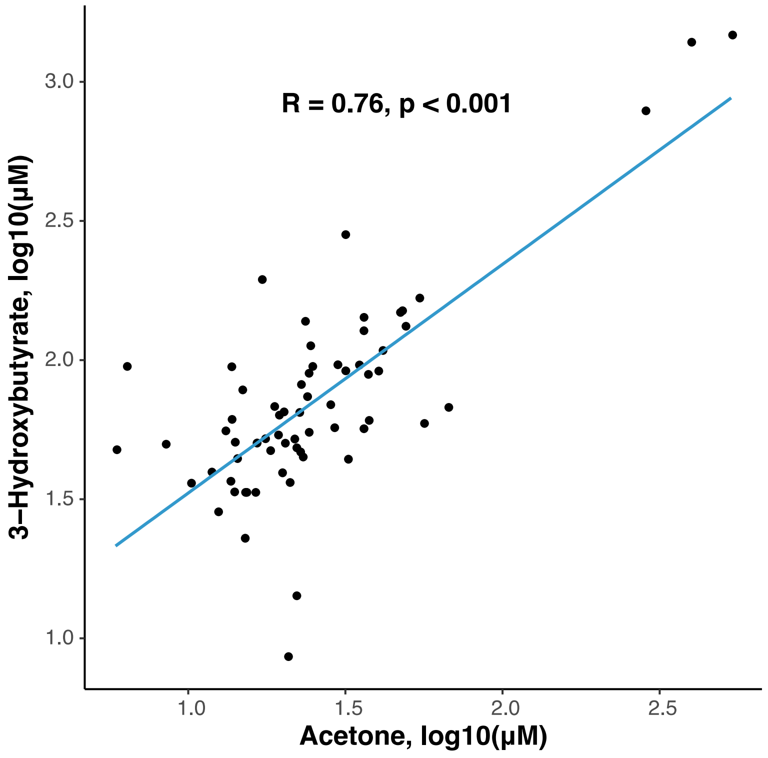

Figure S5. Pearson correlation between 3-hydroxybutyrate and acetone.

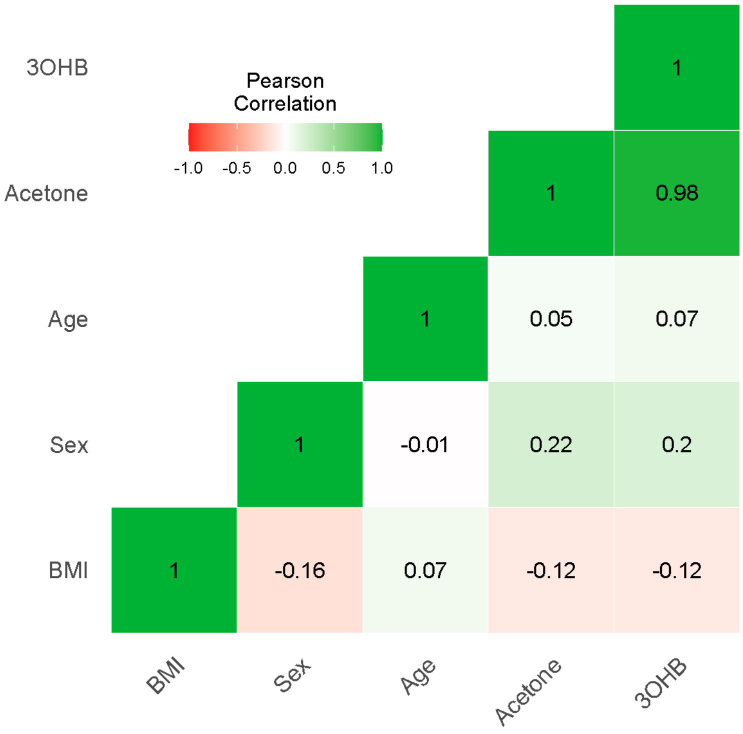

Figure S6. Pearson correlation heatmap analysis of 3-hydroxybutyrate (3OHB), acetone, and anthropomorphic data (age, sex, and body mass index [BMI]).
